# The British Pharmacopœia, 1898

**Published:** 1898-04-16

**Authors:** 


### THE BRITISH PHARMACOPŒIA, 1898.

The new Pharmacopœia has been completed, and so soon as the Treasury can make up its mind as to the price at which it is to be sold it will be issued. As was to be expected, considerable alterations have been made. The metric system of weights and measures has been introduced, many drugs have been omitted, and some have been added. Moreover, a large number of superfluous preparations have been left out, and space has been saved by abbreviating the paragraghs which were more or less descriptive of the sources or modes of preparation of substances found in commerce. In regard to the omissions, we find that most of what may be called kitchen drugs have been left out, such as milk, honey, treacle, vinegar, raisins, &c. Then all the poultices have vanished from the scene. Many preparations also have been removed which really took the form of prescriptions. Thus all the enemata, all the inhalations, and half-a-dozen of the ointments have been removed, among the latter being the elemi, savin, and turpentine ointments, and even the old simple ointment. Just half the confections have gone, viz., those of hips, opium, scammony, and turpentine. The Confectio Rosæ Gallicæ, however, remains as a handy basis for pill masses. Both the old-fashioned decoctions of sarsaparilla with which physicians used to modify in a vague and uncertain manner the therapeutic efficacy of their mercurials have vanished, and in their place we find a concentrated compound solution of sarsaparilla. In fact, of the decoctions only those of aloes, pomegranate, and logwood remain, and no new ones are added. Many drugs and preparations have been removed after a comparatively short life. Thus, solution of dialysed iron, rhamnus frangula bark, fir wood oil, and extract of jaborandi have gone, the last being replaced by a liquid extract. Among the additions we find a pancreatic solution, ointments of capsicum and cocaine, a mercuric oleate ointment, apparently to replace the oleate of mercury which is withdrawn, and physostigmine sulphate instead of physostigmine, which has been removed. A series of concentrated solutions are introduced which will probably be found very useful, taking the place of the concentrated preparations which have for a long time been supplied by the wholesale houses. The following are the drugs so dealt with—sarsaparilla, calumba, chiretta, cusparia, krameria, quassia, rhubarb, senega, senna, serpentary. Effervescing citrates of caffeine and of lithium are added, the effervescing solutions of lithia, potash, and soda being removed. There is a glycerine of boric acid, which we presume is an equivalent of the well-known boroglyceride. Among the new lozenges we find the very useful one containing guaiacum resin, a simple krameria lozenge, and one containing cocaine, and a phenol lozenge. Among the animal substances there are a glycerine of pepsine, a pancreatic solution, a thyroid solution, and dry thyroid substance. Gutta-percha is knocked out and india-rubber is put in. A syrup and a tincture of Virginian prune (which a medical contemporary speaks of as a flavouring agent!) are introduced. Oil of pine, prepared coal tar, quillaia bark, salol, terebene, benzol, discs of homatropine, an infusion of broom instead of the decoction, liquid paraffin, naphthol, a syrup of glucose, an ammoniated tincture of ergot, and lastly, bisulphide of carbon, probably to dissolve the india-rubber, are among the new additions to the Pharmacopœia. It is worthy of note that very few of the newer coal tar derivatives find a place, and that the list of the drugs and preparations added to the Pharmacopœia is much shorter than that of those which have been removed, which may be taken as a sign of the times.

